# Effects of adrenaline and vasopressin on cerebral microcirculation at baseline and during global brain ischemia and reperfusion in rabbits

**DOI:** 10.1186/s12871-022-01926-9

**Published:** 2022-12-08

**Authors:** Daisuke Kondo, Nobumasa Asano, Tadahiko Ishiyama, Noriyuki Shintani, Takashi Matsukawa

**Affiliations:** 1 Department of Anesthesiology, Faculty of Medicine, University of Yamanashi, 1110 Shimokato, Chuo, Yamanashi 409-3898 Japan; 2 Surgical Center, University of Yamanashi Hospital, University of Yamanashi, Chuo, Yamanashi Japan

**Keywords:** Pial arterioles, Adrenaline, Vasopressin, Ischemia and reperfusion

## Abstract

**Background:**

During cardiopulmonary resuscitation, the brain becomes ischemic. Adrenaline and vasopressin have been recommended for use during cardiopulmonary resuscitation. We aimed to investigate the direct effects of adrenaline and vasopressin on the cerebral microvasculature at baseline and during ischemia and reperfusion in rabbits.

**Methods:**

The closed cranial window method was used to visualize the cerebral microcirculation and changes in the pial arteriole diameter in rabbits. Adrenaline and vasopressin were administered topically on the brain tissue. First, the effects of adrenaline and vasopressin on pial arterioles were evaluated in 7 rabbits that were given 4 different concentrations of adrenaline, and another 7 rabbits that received 4 different concentrations of vasopressin. Second, the effects of adrenaline and vasopressin were determined during the global brain ischemia and reperfusion, which was induced by clamping the brachiocephalic, left common carotid, and left subclavian arteries for 15 min. An additional 21 rabbits were randomly assigned to receive artificial cerebrospinal fluid (aCSF) (*n* = 7), adrenaline 10^–5^ mol/L (*n* = 7), or vasopressin 10^–7^ mol/L (*n* = 7). Each drug was continuously infused from 5 min after the initiation of ischemia until 120 min after reperfusion. The pial arteriole diameters were recorded before and during ischemia, and after reperfusion.

**Results:**

At baseline, adrenaline and vasopressin did not affect the cerebral pial arterioles. During ischemia, vasopressin, but not aCSF and adrenaline constricted the pial vessels. Late in the reperfusion phase, pial diameter became reduced in the vasopressin and aCSF groups whereas pial diameter was higher in the animals treated with adrenaline.

**Conclusions:**

Adrenaline and vasopressin did not affect pial arterioles at baseline. During reperfusion, adrenaline may counteract the cerebral vasoconstriction.

## Background

Cardiac arrest causes global brain ischemia and cerebral blood flow remains reduced during cardiopulmonary resuscitation. Adrenaline and vasopressin have been recommended for use during cardiopulmonary resuscitation. Adrenaline shows strong vasoconstrictor action through α-adrenergic stimulation and increases myocardial contractility through β-adrenergic stimulation. In cerebral circulation, pial arterioles are constricted via α-adrenergic stimulation [1, 2], and they are dilated via a β-adrenergic mechanism [3]. It is unclear whether vasoconstriction or vasodilation predominates in the effects of adrenaline on cerebral blood vessels and under which conditions adrenaline causes vasoconstriction or vasodilation [4, 5].

Vasopressin is a potent vasoconstrictor that constricts systemic arteries by the stimulation of V1 vasopressin receptors [6]; however, the effects of vasopressin on the cerebral vessels are controversial. One study showed that vasopressin has a dual effect on arterioles, that is, dilatation at low concentrations and constriction at high concentrations [7]. Another study revealed that vasopressin elicits pial arteriolar dilation [8].

During cardiopulmonary resuscitation, the brain becomes ischemic whereas resuscitation may cause reperfusion injury. After successful resuscitation, cerebral hyperperfusion is induced, followed by hypoperfusion (postischemic delayed hypoperfusion) [9]. During hypoperfusion, the delivery of oxygen and glucose may be impaired which could affect outcome after resuscitation. One study indicated that adrenaline was superior to vasopressin during resuscitation in terms of survival rate [10]; however, when the α2 effects of adrenaline were blocked, adrenaline had adverse effects on cerebral microvascular blood flow through its α1 agonist action [11]. Additionally, we previously reported that pial arterioles constricted during the reperfusion period after 10 min clamping of brachiocephalic artery, left common carotid artery, and left subclavian artery [12]. Further, because α-blockade attenuates cerebral hypoperfusion, an α-stimulant adrenaline may accentuate cerebral hypoperfusion following cerebral ischemia [13]. In cardiopulmonary resuscitation, the degree of brain damage following resuscitation determines a patient's quality of life; therefore, adrenaline is not always preferred from the perspective of brain resuscitation. On the other hand, some studies showed that vasopressin improved vital organ blood flow during resuscitation [14, 15]; furthermore, it increased cerebral microvascular blood flow compared with that of adrenaline after cardiopulmonary resuscitation [16]. Thus, vasopressin may be superior to adrenaline in terms of brain resuscitation.

In this study, we first investigated the direct effects of adrenaline and vasopressin on cerebral arteriolar tone at baseline using the cranial window technique in rabbits, hypothesizing that both adrenaline and vasopressin would induce pial arteriolar constriction. Thereafter, we evaluated the direct effects of adrenaline and vasopressin on the pial arterial diameter changes during the ischemia and reperfusion period and hypothesized that the effects of the two drugs would be different. The primary outcome was the pial arteriolar diameter change at 120 min after unclamping; the secondary outcomes were changes in the parameters of hemodynamic and blood gas analyses.

## Methods

The experimental protocol was approved by the Committee on Animal Research at the University of Yamanashi, Japan. Experiments were performed on 35 young 17–20-week-old Japanese White rabbits weighing between 2.8 and 3.6 kg. The rabbits were provided by the animal experiment center of the University of Yamanashi. After gaining intravenous access via an ear vein, the animals were anesthetized with pentobarbital sodium (20 mg/kg), and anesthesia was maintained with a continuous infusion (5 mg/kg/h). The animals were tracheostomized and mechanically ventilated with 60% oxygen to avoid hypoxemia. End-tidal CO_2_ (EtCO_2_) was monitored; tidal volume and respiratory rate were adjusted to maintain arterial carbon dioxide partial pressure (PaCO_2_) between 35 and 45 mmHg. A catheter was inserted into the femoral artery to measure the mean arterial pressure (MAP) and for blood sampling. Rectal temperature was monitored constantly and maintained at 39 ℃ ± 1 ℃ with a heating blanket because the normal temperature of Japanese White rabbits has been reported to be around 39 ℃ [17, 18]. After the experiment, the rabbits were sedated with 100 mg/kg of pentobarbital sodium and euthanized via an intravenous injection of 10 mL of potassium chloride (2 mEq/mL). Euthanasia was confirmed by the loss of arterial pressure and waveforms of an expiratory CO2 monitor.

### Cranial window installation

Cranial window installation and the ischemia and reperfusion technique were performed in accordance with our previous studies [12, 19]. After undergoing a thoracotomy, the rabbits were placed in the sphinx posture, and a closed cranial window was implanted over the parietal cortex. The diameter of the cranial window was 8 mm, which was placed in the parietal bone. The dura and arachnoid membranes were cut, and a ring with thin glass was then positioned over the hole and secured with bone wax and dental acrylic. The space under the window was filled with artificial cerebrospinal fluid (aCSF), and three polyethylene catheters were inserted; one catheter was attached to a reservoir bottle containing aCSF, which was continuously bubbled with 5% CO_2_ in air. The aCSF was suffused at 0.1 mL/min. Two other catheters served as an inlet and outlet for the aCSF and study drug solutions; the level of each outlet was maintained at approximately 5–6 cm above the window to maintain normal intracranial pressure. The fluid volume in the window was between 0.5 and 0.7 mL. The composition of the aCSF was as follows: Na^+^, 151 mEq/L; K^+^, 3.5 mEq/L; Ca^2+^, 2.5 mEq/L; Mg^2+^, 1.3 mEq/L; HCO_3_^−^, 25 mEq/L; urea, 40 mg/dL; and glucose, 65 mg/dL. At each measurement point, one image of the pial vessels was captured, recorded, and measured using a digital video analyzer (VHX-500F, Keyence, Osaka, Japan) attached to a microscope (VH-Z25, Keyence). The microscopic magnification was × 150. The resolution of the video system was 1600 × 1200 pixels. The measured diameters of the pial arterioles were between 15 and 90 μm.

### Experiment 1 (baseline)

**Fig. 1:**
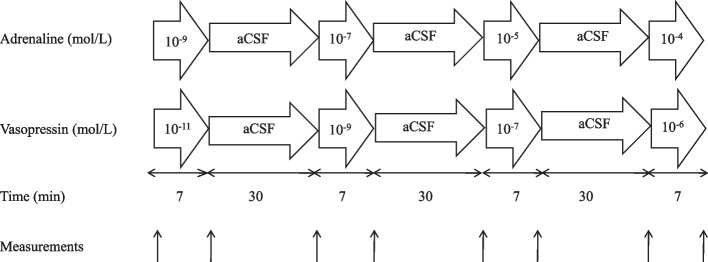
Flow chart of Experiment 1 (baseline)

Fourteen rabbits were divided into two groups to received either adrenaline (*n* = 7) or vasopressin (*n* = 7). After recording the control measurements of the hemodynamic parameters and pial arteriole diameters, the cranial window was superfused with four increasing concentrations of adrenaline (10^–9^, 10^–7^, 10^–5^, and 10^–4^ mol/L dissolved in the aCSF) or vasopressin (10^–11^, 10^–9^, 10^–7^, and 10^–6^ mol/L dissolved in the aCSF). The perfusion rate was initially set at 0.5 mL/min for 2 min, decreasing to 0.1 mL/min for 5 min. The pial arteriole diameter, MAP, heart rate (HR), and rectal temperature were measured at the end of the administration of each concentration. The window was then flushed with aCSF for 30 min before testing the next concentration. Figure 1 shows the flowchart of Experiment 1.

### Experiment 2 (Ischemia and reperfusion)

**Fig. 2:**
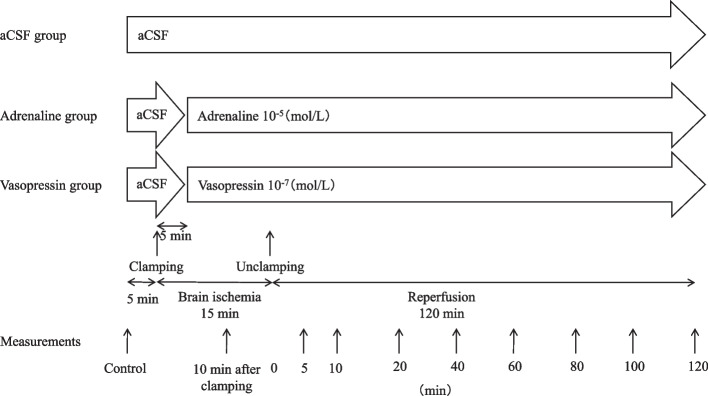
Flow chart of Experiment 2 (Ischemia and reperfusion)

Before the experiments, the brachiocephalic artery, left common carotid artery, and left subclavian artery were exposed. Five min after the control measurements were obtained, global brain ischemia was produced by a 15 min clamp of the brachiocephalic artery, left common carotid artery, and left subclavian artery. Twenty-one rabbits, separate from those in Experiment 1, were divided into three groups of 7 rabbits each. In the aCSF group, aCSF was infused throughout the study period (*n* = 7). Either adrenaline 10^–5^ mol/L (adrenaline group, *n* = 7), or vasopressin 10^–7^ mol/L (vasopressin group, *n* = 7) was infused from 5 min after the initiation of ischemia until up to 120 min following unclamping. Measurements of the cerebral pial diameter, hemodynamic parameters, and arterial blood were made at the following time points: 5 min before ischemia (control); 10 min after clamping; and at 5, 10, 20, 40, 60, 80, 100, and 120 min after unclamping. Figure 2 shows the flowchart of the Experiment 2.

### Statistical analysis

Values are represented as the mean ± SD. Statflex version 6 was used for statistical analysis. The mean changes in vessel diameter at 120 min after reperfusion in previous ischemia and reperfusion studies were 10–20% with a standard deviation of approximately 10% [12, 19]. Power analysis indicated that a sample size of 7 rabbits per group was sufficient to detect a 15% change in the pial arteriolar diameter from the control values with a power of 0.8 and α < 0.05. The differences between control values and each concentration (Experiment 1), and each of the nine time points (Experiment 2) were compared using a two-way analysis of variance (ANOVA) and the Dunnett’s post-hoc test; the differences among the three groups at corresponding time points were evaluated by ANOVA and the Newman–Keuls post-hoc test. A *p-*value less than 0.05 was considered statistically significant.

## Results

### Experiment 1 (baseline)

In the baseline experiments using adrenaline and vasopressin, the MAP, HR, arterial pH, PaCO_2_, partial pressure of oxygen (PaO_2_), base excess (BE), and glucose levels did not change significantly during the experimental period (Table 1).

**Table 1:** Physiologic measurements during topical application of adrenaline at baseline

	MAP (mm Hg)	HR (beats/min)	pH	PaCO_2_ (mm Hg)	PaO_2_ (mm Hg)	BE (mmol/L)	Glucose (mg/dL)
A Control	96 ± 12	299 ± 43	7.44 ± 0.07	39.1 ± 4.8	215 ± 30	2.6 ± 3.0	145 ± 22
A 10^–9^ mol/L	95 ± 6	281 ± 33	7.43 ± 0.05	36.6 ± 3.6	206 ± 25	0.3 ± 2.9	136 ± 15
A 10^–7^ mol/L	99 ± 6	265 ± 32	7.40 ± 0.05	39.1 ± 5.1	204 ± 28	0.9 ± 3.2	132 ± 19
A 10^–5^ mol/L	100 ± 13	286 ± 26	7.43 ± 0.05	39.2 ± 3.6	202 ± 21	2.1 ± 2.8	133 ± 22
A 10^–4^ mol/L	98 ± 9	289 ± 28	7.42 ± 0.03	38.9 ± 2.0	201 ± 28	1.1 ± 3.1	138 ± 28
V Control	95 ± 4	306 ± 39	7.44 ± 0.03	37.3 ± 2.7	202 ± 24	1.9 ± 3.0	145 ± 16
V 10^–11^ mol/L	96 ± 5	308 ± 46	7.43 ± 0.05	39.5 ± 4.1	214 ± 24	2.0 ± 2.5	151 ± 9
V 10^–9^ mol/L	94 ± 4	282 ± 53	7.44 ± 0.04	38.2 ± 4.3	216 ± 26	1.7 ± 2.5	135 ± 13
V 10^–7^ mol/L	92 ± 7	298 ± 48	7.45 ± 0.03	37.8 ± 4.1	204 ± 31	2.4 ± 1.1	135 ± 17
V 10^–6^ mol/L	89 ± 15	285 ± 49	7.43 ± 0.03	39.8 ± 3.1	203 ± 42	1.8 ± 1.6	136 ± 18

Values are mean ± SD

*MAP* Mean arterial blood pressure, *HR* Heart rate, *BE* Base excess, *PaCO*_*2*_ Partial pressure of carbon dioxide, *PaO*_*2*_ Partial pressure of oxygen, *A* adrenaline, *V* Vasopressin

**Fig. 3:**
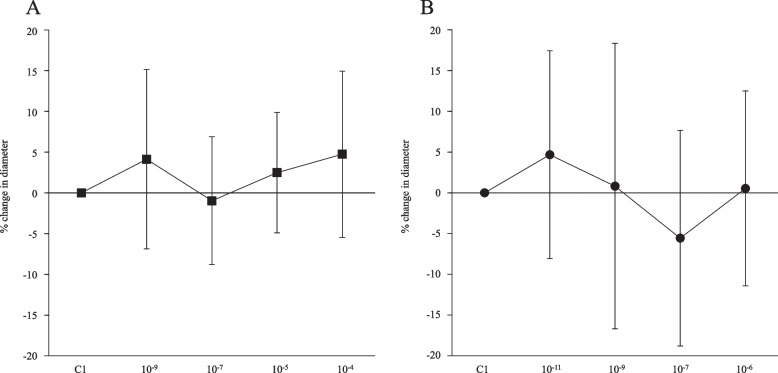
The effects of adrenaline (**A**) and vasopressin (**B**) on the pial arterioles at baseline. Values are mean ± standard deviation. Data represent percent change in diameter in comparison with pre-drug measurements. C1, control for experiment 1

The topical application of adrenaline and vasopressin had no effect on pial arteriole diameter (Fig. 3A and B).

### Experiment 2 (Ischemia and reperfusion)

The MAP, HR, PaCO_2_, and PaO_2_ were unaffected by clamping in all three groups. Unclamping reduced MAP in the aCSF and vasopressin groups while there was a trend towards a reduction in MAP in the adrenaline group (*P* < 0.05) and BE decreased in the adrenaline and vasopressin groups, while the glucose level increased in the adrenaline group. The HR, PaCO_2_, and PaO_2_ did not change significantly in the aCSF, adrenaline, and vasopressin groups during the experimental period (Tables 2, 3, and 4).

**Table 2:** Physiologic measurements during the ischemia and reperfusion in the aCSF group

	MAP (mm Hg)	HR (beats/min)	pH	PaCO_2_ (mm Hg)	PaO_2_ (mm Hg)	BE (mmol/L)	Glucose (mg/dL)
Control	106 ± 13	285 ± 37	7.40 ± 0.05	37.6 ± 2.9	186.6 ± 20.3	-1.9 ± 2.3	145 ± 22
After 10 min of ischemia	105 ± 35	276 ± 24	7.40 ± 0.06	35.0 ± 4.3	165.7 ± 40.5	-2.9 ± 3.1	188 ± 54*
Unclamp 5 min	75 ± 41*	261 ± 22	7.29 ± 0.08*	40.5 ± 5.7	170.0 ± 45.9	-5.4 ± 2.4	172 ± 35
Unclamp 10 min	93 ± 17	271 ± 34	7.31 ± 0.06	41.4 ± 5.7	174.6 ± 45.0	-5.3 ± 2.5	166 ± 39
Unclamp 20 min	94 ± 29	265 ± 34	7.31 ± 0.07	42.2 ± 5.0	161.6 ± 37.4	-5.1 ± 2.7	137 ± 25
Unclamp 40 min	86 ± 15	267 ± 25	7.33 ± 0.06	39.3 ± 2.8	161.0 ± 41.3	-4.1 ± 3.2	137 ± 25
Unclamp 60 min	84 ± 14	266 ± 26	7.32 ± 0.07	39.3 ± 2.8	165.8 ± 47.7	-4.0 ± 4.3	124 ± 21
Unclamp 80 min	89 ± 14	269 ± 26	7.34 ± 0.07	38.0 ± 4.6	158.6 ± 37.0	-2.4 ± 2.6	125 ± 21
Unclamp 100 min	90 ± 13	268 ± 28	7.35 ± 0.09	39.4 ± 3.7	169.4 ± 39.0	-3.3 ± 4.3	123 ± 24
Unclamp 120 min	90 ± 14	268 ± 29	7.33 ± 0.10	39.9 ± 3.4	156.9 ± 35.4	-3.7 ± 5.2	122 ± 28

Values are mean ± SD

*MAP* Mean arterial blood pressure, *HR* Heart rate, *BE* Base excess, *PaCO*_*2*_ Partial pressure of carbon dioxide, *PaO*_*2*_ Partial pressure of oxygen

^*^ *P* < 0.05 compared with the control value

**Table 3:** Physiologic measurements during the ischemia and reperfusion in the adrenaline group

	MAP (mm Hg)	HR (beats/min)	pH	PaCO_2_ (mm Hg)	PaO_2_ (mm Hg)	BE (mmol/L)	Glucose (mg/dL)
Control	108 ± 10	308 ± 22	7.40 ± 0.04	39.8 ± 1.5	178.3 ± 38.8	-0.6 ± 2.8	139 ± 11
After 10 min of ischemia	124 ± 38	259 ± 51	7.33 ± 0.09	35.0 ± 8.4	150.6 ± 52.4	-6.9 ± 3.0^*^	233 ± 34^*^
Unclamp 5 min	76 ± 34	277 ± 57	7.28 ± 0.09*	40.8 ± 5.9	162.4 ± 71.9	-8.4 ± 3.8^*^	228 ± 53^*^
Unclamp 10 min	80 ± 25	280 ± 51	7.29 ± 0.07	40.1 ± 3.5	165.6 ± 51.2	-8.3 ± 3.4^*^	238 ± 66^*^
Unclamp 20 min	94 ± 12	285 ± 40	7.29 ± 0.07	40.0 ± 4.3	164.3 ± 46.8	-9.0 ± 5.1^*^	232 ± 85^*^
Unclamp 40 min	84 ± 13	282 ± 36	7.32 ± 0.09	38.2 ± 2.9	152.7 ± 52.5	-6.9 ± 4.7^*^	212 ± 94*
Unclamp 60 min	79 ± 15	286 ± 38	7.30 ± 0.11	40.4 ± 3.0	162.2 ± 59.2	-6.1 ± 4.3^*^	200 ± 93
Unclamp 80 min	74 ± 21*	290 ± 25	7.33 ± 0.09	38.6 ± 2.8	152.9 ± 63.0	-6.1 ± 4.6^*^	195 ± 98
Unclamp 100 min	76 ± 27	286 ± 21	7.32 ± 0.10	38.0 ± 3.1	141.1 ± 57.8	-6.6 ± 5.9^*^	192 ± 1 05
Unclamp 120 min	78 ± 27	280 ± 11	7.31 ± 0.16	39.7 ± 5.0	151.4 ± 63.6	-6.6 ± 6.8^*^	172 ± 61

Values are mean ± SD

*MAP* Mean arterial blood pressure, *HR* Heart rate, *BE* Base excess, *PaCO*_*2*_ Partial pressure of carbon dioxide, *PaO*_*2*_ Partial pressure of oxygen

^*^ *P* < 0.05 compared with the control value

**Table 4:** Physiologic measurements during the ischemia and reperfusion in the vasopressin group

	MAP (mm Hg)	HR (beats/min)	pH	PaCO_2_ (mm Hg)	PaO_2_ (mm Hg)	BE (mmol/L)	Glucose (mg/dL)
Control	109 ± 18	278 ± 44	7.40 ± 0.03	39.6 ± 4.0	200.0 ± 36.2	-0.6 ± 1.9	138 ± 15
After 10 min of ischemia	103 ± 37	290 ± 64	7.36 ± 0.06	37.3 ± 8.7	166.0 ± 79.2	-4.3 ± 3.3*	199 ± 39
Unclamp 5 min	75 ± 29*	269 ± 44	7.31 ± 0.06^*^	41.6 ± 3.0	179.7 ± 60.3	-5.4 ± 3.5^*^	199 ± 57
Unclamp 10 min	78 ± 30*	265 ± 31	7.31 ± 0.07^*^	41.4 ± 5.1	183.4 ± 60.4	-5.4 ± 3.7^*^	197 ± 71
Unclamp 20 min	81 ± 29	266 ± 33	7.31 ± 0.06^*^	42.1 ± 6.3	191.0 ± 35.4	-5.0 ± 4.0*	196 ± 101
Unclamp 40 min	91 ± 28	275 ± 27	7.33 ± 0.07	41.5 ± 3.6	182.3 ± 31.6	-4.0 ± 3.5	195 ± 121
Unclamp 60 min	89 ± 24	287 ± 30	7.35 ± 0.06	39.9 ± 3.9	183.4 ± 18.6	-4.0 ± 2.9	187 ± 89
Unclamp 80 min	90 ± 31	290 ± 34	7.34 ± 0.06	40.0 ± 4.7	158.0 ± 27.5	-4.0 ± 2.6	187 ± 80
Unclamp 100 min	92 ± 27	287 ± 33	7.35 ± 0.05	40.1 ± 4.3	156.3 ± 27.4*	-3.4 ± 2.4	181 ± 75
Unclamp 120 min	88 ± 28	284 ± 36	7.37 ± 0.06	39.5 ± 5.1	166.1 ± 36.7	-2.7 ± 3.1	174 ± 73

Values are mean ± SD

*MAP* Mean arterial blood pressure, *HR* Heart rate, *BE* Base excess, *PaCO*_*2*_ Partial pressure of carbon dioxide, *PaO*_*2*_ Partial pressure of oxygen

^*^P < 0.05 compared with the control value

**Fig. 4:**
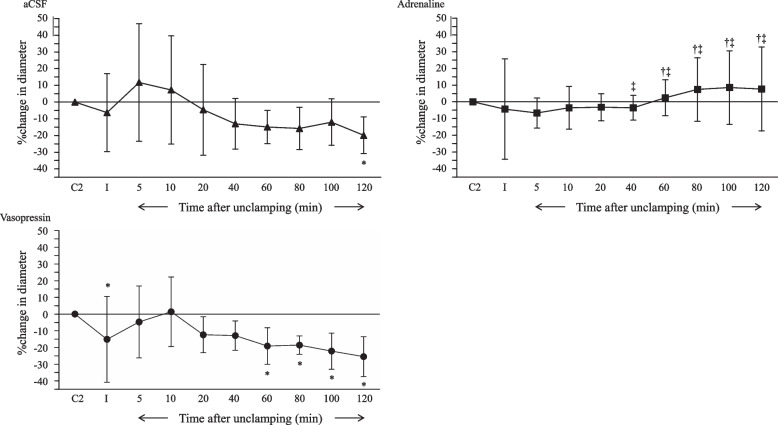
The effects of aCSF, adrenaline, and vasopressin on pial arterioles during the global brain ischemia and reperfusion period. Values are mean ± standard deviation. Data represent the percent change in diameters from the baseline values. aCSF, artificial cerebrospinal fluid; C2, control for experiment 2; I, 10 min after clamping. * *p* < 0.05 compared with control, † *p* < 0.05 compared with aCSF, ‡ *p* < 0.05 compared with vasopressin

**Fig. 5:**
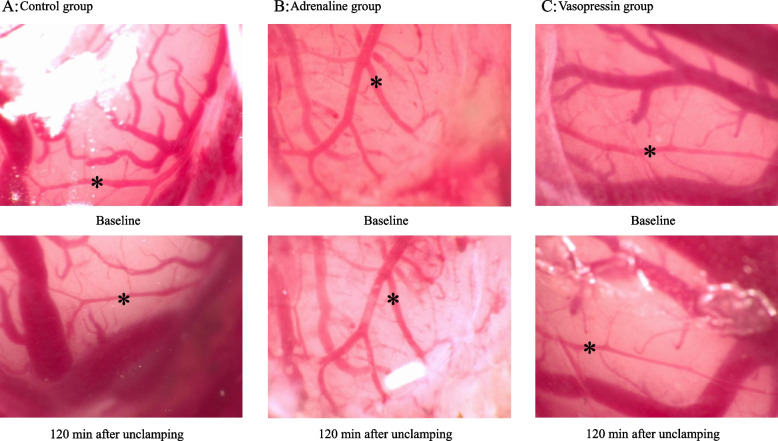
Representative images of the pial microvessels before and 120 min after ischemia and reperfusion. * pial arteriole

Pial arteriolar diameter was unaffected by clamping in the aCSF and adrenaline groups, whereas diameter decreased in the vasopressin group. The pial arteriolar constriction was observed after 120 min of unclamping in the aCSF group (*p* < 0.05, Fig. 4A). The pial arterioles were not affected during ischemia and reperfusion in the animals that received adrenaline, but the diameter was greater than that in the other groups from 60 to 120 min after unclamping (Fig. 4B). In the vasopressin group, the pial arteriolar diameter decreased at 60 to 120 min after unclamping (*p* < 0.05, Fig. 4C). There was no difference between the aCSF and vasopressin groups in arteriolar diameter (Fig. 4A and C). Representative images of the pial microvessels before and 120 min after ischemia and reperfusion are shown in Fig. 5.

## Discussion

This study showed that adrenaline and vasopressin at both low and high doses had no direct effects on the pial arterioles at baseline. During ischemia, pial vessels were constrictied by vasopressin and unaffected in the animals that received aCSF and adrenaline, whereas the vessels constricted during late reperfusion in the vasopressin group and only at the end of the experiment in the aCSF group. Pial diameter was maintained throughout the reperfusion period in the animals that received adrenaline, and adrenaline resulted in greater pial diameter from 60 to 120 min after unclamping as compared to aCSF and vasopressin. Conversely, vasopressin acted on pial arterioles similarly to aCSF during the reperfusion period, but the diameter decreased below the control level from an earlier reperfusion time point in the vasopressin group.

The conflicting effects of adrenaline on cerebral arterioles have been reported previously [4, 5]. One study showed that adrenaline constricted the cerebral pial arterioles in cats [4]. Another study suggested that adrenaline did not affect the cerebral pial arterioles in dogs [5]. Our findings concur with the results of the latter study and indicated that adrenaline did not dilate nor constrict the pial arterioles; thus, our study suggests that adrenaline could act on α1-, α2-, β1-, and β2-receptors to a comparable extent in rabbits at baseline.

Various studies have suggested that vasopressin induces vasodilation [20] or vasoconstriction [21]. Vasopressin stimulates the production of nitric oxide from vascular endothelial cells through V1- and V2-receptors [22, 23]. Through this mechanism, vasopressin dilates the cerebral pial arterioles [23]; conversely, vasopressin can also constrict the cerebral vessels through a V1-receptor mediated mechanism [23]. Kumazawa et al. [7] reported that the pial arterioles were dilated at lower doses (10^–11^ mol/L) of vasopressin, whereas they were constricted at higher doses (10^–9^, 10^–7^, and 10^–5^ mol/L). Takayasu et al. [23] demonstrated that vasopressin induced a triphasic response (vasodilation, vasoconstriction, and vasodilation) in these arteries. In contrast, we found that vasopressin did not affect the pial arterioles at baseline.

We used adrenaline at 10^–5^ mol/L for the ischemia and reperfusion study. The plasma adrenaline concentration during cardiopulmonary resuscitation reportedly varies between 5 × 10^–7^ [24] and 1.2 × 10^–6^ mol/L [25] or between 0.124 [26] and 1 μg/mL [27]. As the molecular weight of adrenaline is 183.2 g/mol, adrenaline at 10^–5^ mol/L is equivalent to 1.832 μg/mL, a relatively higher concentration. However, in the cranial window technique, the drug solution injected into the window is slightly diluted by the cerebrospinal fluid of the animal; therefore, the adrenaline concentration of 10^–5^ mol/L should be clinically significant. In the ischemia and reperfusion study, the vasopressin dose was 10^–7^ mol/L. Plasma vasopressin concentration during cardiopulmonary resuscitation reportedly ranges from 70,000 [28] to 110,000 pg/mL [29]. The molecular weight of vasopressin is 1084.2316 g/mol. Thus, vasopressin concentrations of 70,000 and 110,000 pg/mL are approximately equal to 6.5 × 10^–8^ and 10^–7^ mol/L, respectively; therefore, a vasopressin concentration of 10^–7^ mol/L should also be clinically significant.

Catecholamines may be used clinically to maintain hemodynamics after successful resuscitation. Moreover, in a previous global brain ischemia and reperfusion study, we reported that pial arterioles temporarily dilated and subsequently constricted pial arterioles throughout the reperfusion period [12, 19]. Adrenaline and vasopressin were continuously administered in this study to elucidate their effects on cerebral vasoconstriction during reperfusion following global cerebral ischemia.

Postischemic delayed hypoperfusion reportedly contributes to the development of cerebral edema [30]; similarly, preventing cerebral vasoconstriction following ischemic stroke has been shown to improve long-term neurological outcomes in female rats [31]. Cerebral vasodilation following brain ischemia might remove acidic metabolites from the ischemic brain tissue. Additionally, cerebral vasodilation may provide sufficient oxygen and glucose to preserve normal neuronal function. Thus, the attenuation of cerebral vasoconstriction following brain ischemia is important, though cerebral hyperemia may also be an issue during resuscitation after cardiac arrest [32]. The current study showed that adrenaline increased the pial arteriolar diameter during the reperfusion period and may counteract cerebral vasoconstriction during the reperfusion period. Thus, adrenaline may be useful during reperfusion whereas it appears to have no effect on the cerebral vasculature during brain ischemia.

Ristagno et al. reported that vasopressin maintained cortical microcirculatory blood flow after cardiac arrest until 6 min after spontaneous circulation was restored [16]. Our results showed that the pial arteriolar diameters at 5 and 10 min following reperfusion were comparable with those before brain ischemia in the vasopressin group. Thus, vasopressin might not deteriorate the cerebral circulation in the early phases of the reperfusion period. However, we found that vasopressin reduced pial arteriolar diameter during ischemia and from 60–120 min after reperfusion. These results might coincide with the findings of the study by Kumazawa et al., which showed that vasopressin constricted the cerebral pial arterioles during the ischemia and reperfusion period [7]. Vasopressin may impair cerebral circulation from 60 min after reperfusion.

Ristagno et al. [16] also reported the effects of adrenaline on the cerebral microcirculatory flow during and after cardiopulmonary resuscitation. They found that adrenaline markedly decreased cerebral cortical microcirculatory blood flow for up to 6 min following the restoration of circulation. In contrast, adrenaline did not constrict the pial arterioles at 5 min following reperfusion in the present study. There are several differences between the study by Ristagno et al. and our study, such as the induction of brain ischemia (cardiac arrest vs arterial clamping), injection site of adrenaline (right atrium vs topical application), vessel diameter (< 20 µm vs 15–90 µm), and animal model(pigs vs rabbits); these differences may have affected the results. Compared to the aCSF and vasopressin groups, we found that the adrenaline group showed dilation of the pial arterioles 40 min following reperfusion. Our study suggested that adrenaline improved cerebral circulation during reperfusion.

This study has some limitations. First, we administered adrenaline and vasopressin into the cranial window to elucidate the direct pharmacological effects of both drugs. The systemic effects of adrenaline and vasopressin could be avoided by topical application; however, our results may not be directly applicable to these clinical situations. Second, cerebral blood flow was not measured in this study; according to Poiseuille’s law, cerebral blood flow should be proportional to the vessel radius. Thus, it is possible that the cerebral blood flow increases and decreases with changes in the arteriolar diameter. Third, the results may have been influenced by the type of anesthesia used. Fourth, this animal model of cerebral ischemia differs from an actual cardiac arrest as the heart was not stopped and the rest of the body was perfused continuously. Further, there was no low-flow period as during cardiopulmonary resuscitation prior to the return of spontaneous circulation.

## Conclusions

In conclusion, adrenaline and vasopressin did not act directly on the pial arterioles at baseline. In the ischemia and reperfusion period, adrenaline increased the cerebral pial arterial diameter at 60, 80, 100, and 120 min after reperfusion compared to those in the vasopressin and aCSF groups. Adrenaline may thus counteract cerebral vasoconstriction during the reperfusion period, whereas vasopressin may constrict pial vessels during cerebral ischemia.

## Data Availability

The datasets generated and/or analyzed during the current study are not publicly available due written in Japanese but are available from the corresponding author on reasonable request.
